# Rapid Estimation of Earthquake Fatalities in Mainland China Based on Physical Simulation and Empirical Statistics—A Case Study of the 2021 Yangbi Earthquake

**DOI:** 10.3390/ijerph19116820

**Published:** 2022-06-02

**Authors:** Yilong Li, Zhenguo Zhang, Wenqiang Wang, Xuping Feng

**Affiliations:** 1 Department of Earth and Space Sciences, Southern University of Science and Technology, Shenzhen 518055, China; 11930700@mail.sustech.edu.cn (Y.L.); 11849528@mail.sustech.edu.cn (W.W.); 11930721@mail.sustech.edu.cn (X.F.); 2 Southern Marine Science and Engineering Guangdong Laboratory (Guangzhou), Guangzhou 511458, China; 3 Guangdong Provincial Key Laboratory of Geophysical High-Resolution Imaging Technology, Southern University of Science and Technology, Shenzhen 518055, China

**Keywords:** earthquake disaster, earthquake fatalities, rapid estimation, earthquake relief, disaster assessment, earthquake emergency response, numerical simulation, empirical method, Yangbi earthquake

## Abstract

At present, earthquakes cannot be predicted. Scientific decision-making and rescue after an earthquake are the main means of mitigating the immediate consequences of earthquake disasters. If emergency response level and earthquake-related fatalities can be estimated rapidly and quantitatively, this estimation will provide timely, scientific guidance to government organizations and relevant institutions to make decisions on earthquake relief and resource allocation, thereby reducing potential losses. To achieve this goal, a rapid earthquake fatality estimation method for Mainland China is proposed herein, based on a combination of physical simulations and empirical statistics. The numerical approach was based on the three-dimensional (3-D) curved grid finite difference method (CG-FDM), implemented for graphics processing unit (GPU) architecture, to rapidly simulate the entire physical propagation of the seismic wavefield from the source to the surface for a large-scale natural earthquake over a 3-D undulating terrain. Simulated seismic intensity data were used as an input for the fatality estimation model to estimate the fatality and emergency response level. The estimation model was developed by regression analysis of the data on human loss, intensity distribution, and population exposure from the Mainland China Composite Damaging Earthquake Catalog (MCCDE-CAT). We used the 2021 Ms 6.4 Yangbi earthquake as a study case to provide estimated results within 1 h after the earthquake. The number of fatalities estimated by the model was in the range of 0–10 (five expected fatalities). Therefore, Level IV earthquake emergency response plan should have been activated (the government actually overestimated the damage and activated a Level II emergency response plan). The local government finally reported three deaths during this earthquake, which is consistent with the model predictions. We also conducted a case study on a 2013 Ms7.0 earthquake in the discussion, which further proved the effectiveness of the method. The proposed method will play an important role in post-earthquake emergency response and disaster assessment in Mainland China. It can assist decision-makers to undertake scientifically-based actions to mitigate the consequences of earthquakes and could be used as a reference approach for any country or region.

## 1. Introduction

Earthquakes are among the most severely damaging natural disasters on Earth. This is especially true in China, where many catastrophic earthquakes have occurred in recent history, such as the 1976 Tangshan earthquake that caused ~242,000 deaths and the 2008 Wenchuan earthquake that caused ~69,000 deaths [1]. At present, no technology can accurately predict earthquakes. Therefore, facilitating timely, scientific judgments on the disaster situation can play an important role in relief after an earthquake. Rapidly estimating the number of fatalities and identifying the earthquake emergency response level can help decision-makers take scientifically-based actions to optimize the earthquake relief plan.

Current earthquake fatality estimation methods can be divided into two categories: analytical and empirical methods [2]. The former requires a variety of detailed data, including building inventories, building structure types, vulnerabilities, and building occupancy. After the earthquake, based on the seismic intensity distribution and the detailed list of the buildings on it, and further combining the damage vulnerability and human loss ratio of different structural types of buildings under different intensities to calculate the number of fatalities in all buildings [3,4,5]. In contrast, empirical methods use seismic parameters (e.g., seismic intensity, magnitude, or ground motion parameters) and demographic data (e.g., population density and number of destroyed buildings) to estimate the number of fatalities from previous earthquakes via statistical regression [2,6,7,8,9,10]. Generally, analytical methods are more accurate than empirical methods. However, implementing an accurate analytical process across entire Mainland China is not feasible. First, high-resolution databases of building characteristics (e.g., building inventory and types) do not currently exist nationwide. Second, Daniell et al. pointed out that >30% of all earthquake fatalities in the Asia Pacific are typically caused by non-structural damage (e.g., heart disease and fire) [11]. Third, the inherent dynamic characteristics of these databases (e.g., the inventory, type, and vulnerability of buildings, which change with societal development), lead to great uncertainty in rapid post-earthquake fatality estimation. Therefore, to rapidly obtain such an estimate after an earthquake in Mainland China, we opted to rely on an empirical method. Li et al. developed an earthquake fatality estimation model based on the Mainland China Composite Damaging Earthquake Catalog (MCCDE-CAT) [1,12]. Based on many earthquake events estimations, Li et al. verified that the model has an optimal evaluation ability for large earthquakes that resulted in tens of thousands of fatalities, such as the Wenchuan earthquake, and small earthquakes that resulted in relatively few fatalities [12].

This estimation model mainly relies on data of seismic intensity distribution and population exposure. Many high-precision population datasets are freely available on the Internet, such as CIESIN [13], Asiapop [14], and LandScan [15]. Three methods are commonly used to obtain seismic intensity data. The first relies on field surveys by earthquake administration. Professionals collect information on damages in a sampling survey unit. By calculating the average damage, the seismic intensity can be estimated according to the predefined Seismic Intensity Scale [16,17]. However, it relies on a limited number of sample sites. When investigators finally draw the seismic intensity distribution map, an elliptical distribution in space is generally assumed. This shape does not reflect the irregular pattern of seismic intensity resulting from differences in underground stress, rock lithology, site effects, and topography. In addition, the investigation process requires substantial human and material resources, and can last several days. Therefore, earthquake intensity cannot be used for the rapid estimation of post-earthquake fatalities. The second (empirical) method relies on the widely used Ground Motion Prediction Equations (GMPEs) [18,19,20]. However, it suffers from several limitations, such as the ergodic hypothesis, insufficient near-source observation data, and insufficient spatial correlation processing [21]. In addition, the statistical and approximate nature of this empirical method hinders appropriate characterization of heterogeneities in seismic intensity, as in the first method.

With the rapid development of computer technology, the third method, namely a large-scale parallel fast numerical calculation method based on physical simulation, has gained significant attention [22,23,24]. This method can simulate the entire physical process of seismic wavefield propagation from the source to the surface. It can calculate the influence of near-surface site effects, terrain, and other factors on the results, thus, producing a detailed spatial distribution of surface seismic intensity. This is a major improvement compared to the two aforementioned methods. Therefore, we selected this method to obtain the seismic intensity distribution, specifically using the three-dimensional (3-D) curved grid finite difference method (CG-FDM) [25,26,27] based on a parallel algorithm [24]. This method is characterized by high calculation speed and high accuracy and can reproduce complex undulating surfaces.

In the following sections of the paper, we first introduce the physically-based numerical simulation method and the earthquake fatality assessment model for Mainland China. Subsequently, considering the Ms 6.4 Yangbi earthquake that occurred in Yunnan Province on 21 May 2021 as an example, we present the results of a rapid assessment of the fatalities after the earthquake. Finally, the proposed method and its features are discussed, followed by concluding comments.

## 2. Methods

The proposed method combines the 3-D CG-FDM with an earthquake fatality estimation model based on regression analysis. The numerical simulation is solved by a GPU owing to its high computational performance. 

### 2.1. Strong Ground Motion Numerical Simulation 

As aforementioned, the 3-D CG-FDM [25,26] was selected as the strong ground motion numerical simulation method for calculating the seismic intensity. The method was selected considering its (1) high calculation accuracy, (2) high calculation efficiency, (3) flexible division of the calculation grid according to the undulating terrain, (4) use of the traction mirror method to calculate free surface, and (5) easy parallel computing. The method has been verified in seismic numerical simulations at various dimensions [25,26,27,28] and has been applied to strong ground motion numerical simulations and rapid seismic disaster analyses [29,30,31,32]. Considering that the theoretical derivation and application of this method have been explained comprehensively in the cited literature, here, we only provided a brief introduction.

The first-order velocity-stress equations for the wave equation in elasticity were as follows:

(1) $$\rho v_{i,t}=\sigma_{ij,j}+f_{i}\;\mathrm{and}$$

(2) $$\sigma_{ij,j}=\lambda \delta_{ij}v_{k,k}+\mu (v_{i,j}+v_{j,i}),$$

where ρ is density, f is the source term, $v_{i}$ presents the velocity component, $\sigma_{ij}$ presents the stress component, λ and μ are the Lamé constants, and $\delta_{ij}$ is the Kronecker tensor.

**Figure 1:**
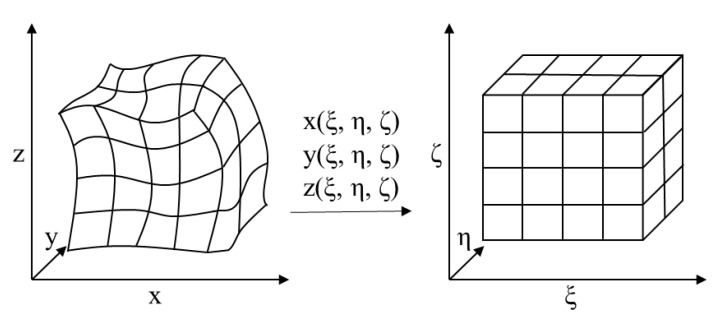
Mapping between (**left**) the curved grid in the physical space (x, y, z) and (**right**) the uniform grid in the computational space (ξ, η, ζ).

To simulate complex surface conditions, the physical quantities in Equations (1) and (2) were mapped from the curved grid in the physical space (x, y, z) to the uniform grid in the computing space (ξ, η, ζ), as shown in Figure 1.

During the specific implementation of the numerical calculation, for integration in the time domain, we adopted the fourth-order Runge–Kutta integral form. When calculating the difference in the spatial domain, the forward and backward difference operators in the DRP/opt MacCormark scheme [33] were used alternately to solve the velocity-stress in Equations (1) and (2) in the Runge–Kutta time marching scheme. Taking the x-axis derivative as an example, the operator forms were as follows:

(3) $$L_{x}^{F}{\left(U\right)}_{i}=\frac{1}{\Delta x}\sum_{n=-1}^{3}a_{n}U_{i+n}\;\mathrm{and}$$

(4) $$L_{x}^{B}{\left(U\right)}_{i}=\frac{1}{\Delta x}\sum_{n=-1}^{3}-a_{n}U_{i-n},$$

where U is a velocity-stress vector [$U={\left(v_{x},v_{y},v_{z},\sigma_{xx},\sigma_{yy},\sigma_{zz},\sigma_{xy},\sigma_{xz},\sigma_{yz}\right)}^{T}$]; $L_{x}^{}$ represents the spatial difference in the x-direction; superscripts *F* and *B* represent the forward and backward difference operators, respectively; *i* represents the grid index; and $a_{n}$ are a set of difference coefficients. For a comprehensive derivation and application of the 3-D CG-FDM, see [25,26].

To rapidly calculate large-scale natural earthquakes, we must also adopt parallel calculation. The graphics processing unit (GPU) has strong parallel computing power, high memory access bandwidth, and low latency [34]. Wang et al. achieved a parallel operation of the CG-FDM and developed a platform for rapid response to earthquake disasters (CGFDM3D-EQR) based on a heterogeneous CPU/GPU architecture [24]. After the earthquake, the platform can rapidly estimate the seismic intensity distribution through parallel numerical simulation within 30 min. To verify the reliability and effectiveness of the platform, they did four numerical simulations and compared them with the instrument observation results, and obtained the expected results. For the case in this paper, we use this computing platform to carry out the strong ground motion numerical simulation.

### 2.2. Earthquake Fatality Estimation Model 

**Figure 2:**
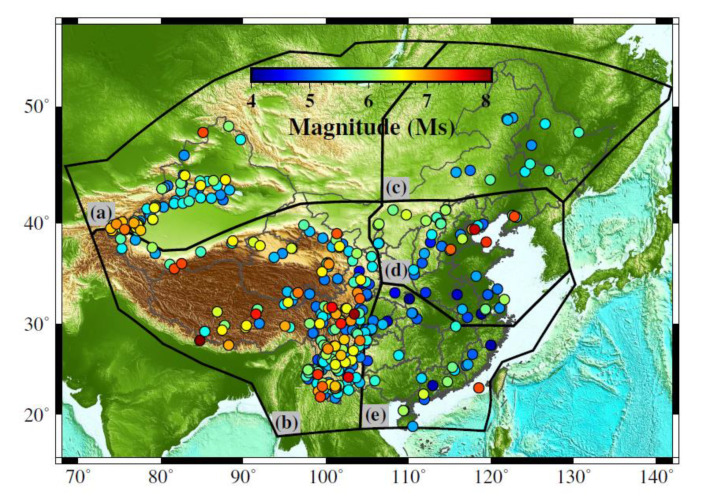
Location of the 377 earthquake epicenters in the Mainland China Composite Damaging Earthquake Catalog (MCCDE-CAT) used by Li et al. to develop the fatality assessment model [12]. The thick black lines divide Mainland China into the five sub-regions as follows (**a**): Xinjiang region, (**b**): Qinghai-Tibet Plateau region, (**c**): Northeast region, (**d**): North China region, and (**e**): South China region.

We used the Mainland China earthquake fatalities estimation model developed by Li et al. [12], which is based on data of seismic intensity and losses for historical earthquakes. Clearly, the performance of this model depends on the completeness of data, and their integrity and consistency across events. Li et al. compiled an open-source MCCDE-CAT (see Data Availability Statement) from 1950 to 2018 based on six earthquake catalogs. They used a large number of previous studies, reports, and websites to verify and complement the information [1]. The catalog includes basic seismological data, social and economic loss data, population exposure data, and intensity distribution data for each earthquake event. Based on data recorded for the 377 earthquake events in the MCCDE-CAT (Figure 2), Li et al. used a logarithmic linear fatality ratio function with two free parameters to develop a set of earthquake fatality estimation models via regression analysis [12]. A large number of earthquake cases were used to verify that the model could estimate >85% of the values within an order of magnitude of the recorded values. A brief introduction to this method is provided below.

According to the characteristics of the geological structures, historical seismicity, focal mechanisms, and social and economic development in Mainland China, the MCCDE-CAT was divided into five sub-regions (as divided by thick black lines in Figure 2). Moreover, according to the differences in the earthquake fatality ratio with time, related to changes in the seismic resistance of buildings, public education, and other factors, the human development index (HDI) was used to evaluate changes in social development level over time to adjust the fatality ratio.

The earthquake-related fatalities were calculated as follows:

(5) $$E=\sum_{I=V}^{\mathrm{XI}}\left(r(I)\times \frac{HDI_{\max-year}}{HDI_{event-year}}\right)\times {\mathrm{Pe}}_{\left(\mathrm{region},\;\mathrm{intensity}=I\right)}$$

where E represents the estimated value, r(I) is the fatality ratio function (which is a logarithmic linear model, i.e., log(r)=β+θ×I, with two free parameters *(β, θ*)), *I* is the seismic intensity. Intensity V-XI is the minimum and maximum intensities of all known seismic records of damaging earthquakes in Mainland China. *HDI* represents the human development index, which is the correction term for the fatality ratio, r, and ${\mathrm{Pe}}_{\left(\mathrm{region},\;\mathrm{intensity}=I\right)}$ represents the number of people exposed in regions with different seismic intensities, *I*.

The two parameters *β* and *θ* of the fatality function were obtained by minimizing the objective function as follows:

(6) $$\varepsilon =\ln\left[\sqrt{\frac{1}{N}\sum_{i=1}^{N}{\left(E_{i}-O_{i}\right)}^{2}}\right]+\sqrt{\frac{1}{N}\sum_{i=1}^{N}{\left[\ln\left(\frac{E_{i}}{O_{i}}\right)\right]}^{2}},$$

where *O_i_* represents the recorded fatalities for the *i*th earthquake event, *E_i_* represents the estimated fatalities in Equation (5), and *N* represents the number of earthquake events.

According to the relevant provisions in the National Earthquake Emergency Plan (NEEP) (see Data Availability Statement) for Mainland China, the Chinese government classifies the earthquake emergency response into four levels as follows: Level IV (≤10 fatalities), Level III (10 < Number of fatalities ≤ 50), Level II (50 < Number of fatalities ≤ 300), and Level I (>300 fatalities). To rapidly evaluate the response level after an earthquake, we constructed a normal cumulative distribution function based on the estimated fatalities and fitting residual calculated using this method (its rationality has been verified using the normality test [12]). The probability *P* of a specific fatality range was calculated as follows:

(7) $$P\left(a<\mathrm{fatality}\leq b\right)=\Phi \left[\frac{\ln b-\ln(E_{i})}{\zeta }\right]-\Phi \left[\frac{\ln a-\ln(E_{i})}{\zeta }\right],$$

where Φ represents the normal cumulative distribution function with respect to the expected value, ln(*E_i_*), and the log residual, ζ. For more details on the development and validation of this estimation model, see [12].

## 3. Case Study: 2021 Ms 6.4 Yangbi Earthquake

Using the above rapid earthquake fatality estimation method, we rapidly calculated the seismic intensity distribution, estimated a total of five deaths, and determined an earthquake emergency response of Level IV within 1 h after the 2021 Ms 6.4 Yangbi earthquake. Later, the government reported that there were three fatalities during this earthquake, consistent with our assessment expectations. This case study of the 2021 Yangbi earthquake is described in detail below.

### 3.1. Background and Data

**Figure 3:**
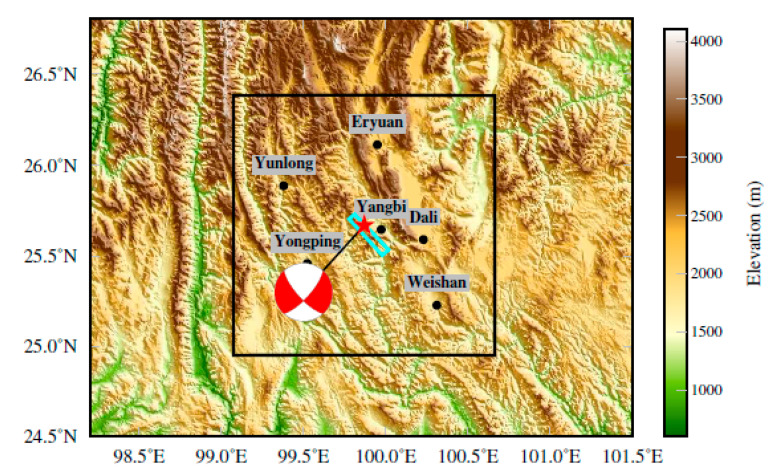
The terrain of the Yangbi earthquake area and its vicinity. The black wireframe represents the calculation region of the strong ground motion simulation. The blue wireframe represents the projection of the fault on the surface. The red star represents the projection of the epicenter onto the surface. The beach ball represents the focal mechanism solution.

At 21:48:34 on 21 May 2021, a Ms 6.4 earthquake occurred in Yangbi County, Dali Bai Autonomous Prefecture, Yunnan Province. The epicenter was located at 99.87° E, 25.67° N, and the focal depth was 8 km. The maximum intensity on the surface was VIII. The earthquake area was located near the southwest boundary of the Chuan-Dian block, where historically strong earthquakes have been relatively frequent. In addition, this region features complex mountains and gullies, rendering it difficult to perform post-earthquake relief work. Figure 3 shows the topographic distribution of the area near the Yangbi earthquake. The overall trend of the mountains in the area is northwest-southeast and the fault trend is similar. The black wireframe in Figure 3 represents the numerical simulation area. The blue wireframe is the projection of the fault on the surface while the red star is the projection of the epicenter onto the surface. The focal mechanism solution shows that this earthquake was a strike-slip earthquake with a large-dip angle (78.3° or 72.9°). 

The 3-D terrain data were extracted from the Shuttle Radar Topographic Mission digital elevation dataset (see Data Availability Statement) provided by the Consultative Group for International Agricultural Research-Consortium for Spatial Information [35]. This dataset is based on the NASA data processed through an interpolation technique to obtain global seamless connection elevation data with a resolution of 90 m. The 3-D medium model used in the simulation was the Chinese crust-upper mantle seismic reference model [36] (see Data Availability Statement). This model is based on data from >2000 seismic stations obtained through environmental noise Rayleigh wave tomography and seismic tomography, with a lateral resolution of 0.5° × 0.5° and a maximum vertical depth of 150 km, covering the entire territory of China. The source data (see Data Availability Statement) were provided by Wang et al., the Institute of Qinghai-Tibet Plateau Research, and the Chinese Academy of Sciences [37].

### 3.2. Numerical Simulation Results 

**Figure 4:**
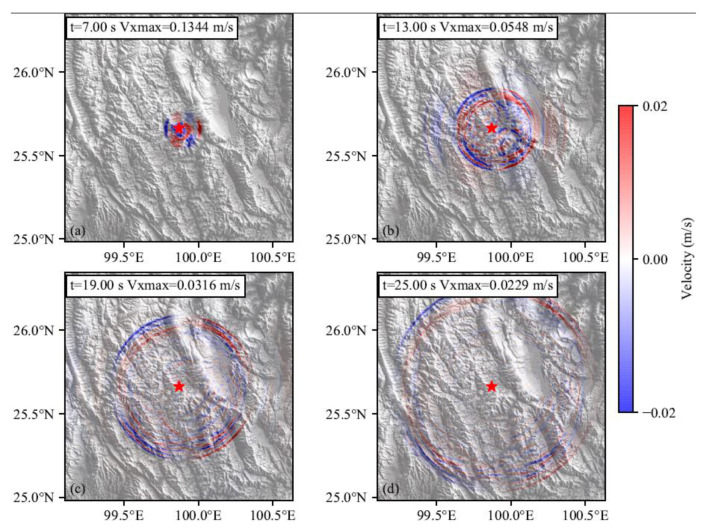
Evolution process of wavefield propagation (east-west velocity component, Vx) along the ground surface at the indicated time points for the Yangbi earthquake. (Note: the color range from –0.02 to 0.02 m/s is not the range of the Vx amplitude. The range was reduced to show the details of ground motion more clearly).

The Yangbi earthquake was numerically simulated using the GPU-based 3-D CD-FDM described in Section 2.1 and the input data described above. The grid size was set to 800 × 800 × 400 and the spatial resolution was 200 m × 200 m. Figure 4 shows four wavefield snapshots during the propagation of the seismic wavefield obtained via numerical simulation. The energy of this earthquake mainly spread outward along the fault strike and perpendicular to the fault strike. Energy in the other directions was weak, consistent with the relatively weak disaster situation in major cities to the east of the epicenter. In addition, the propagation of the seismic wavefield on the surface was complex. Abundant multiple reflection waves were generated near the peaks and ridges along the fault strike; coherent superposition enhanced the ground motion. These simulation results were consistent with those of previous studies on the impact of topography on seismic wavefield propagation [30,38,39,40,41].

**Figure 5:**
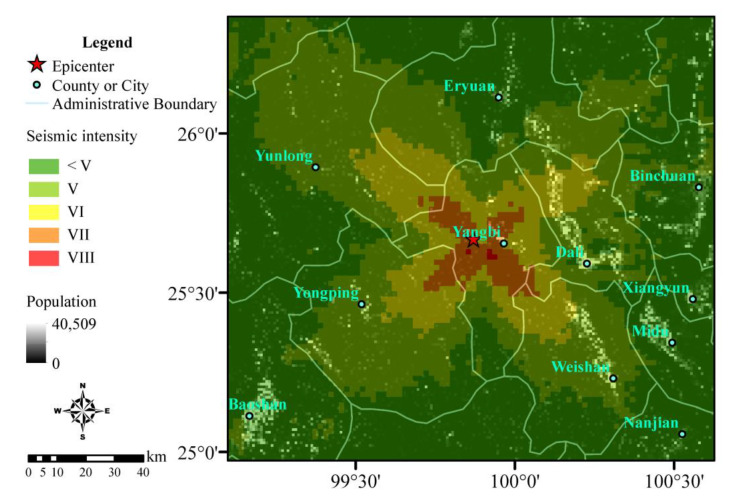
Seismic intensity distribution was obtained via rapid numerical simulations of the Yangbi earthquake. The base map represents the population distribution provided by LandScan [15].

Based on GPU parallel computing, we calculated the seismic intensity distribution according to the PGV-intensity relationship provided by the latest China Seismic Intensity Scale (GB/T 17742-2020) (see Data Availability Statement), as shown in Figure 5, where the base map represents the population distribution data (see Data Availability Statement) provided by LandScan [15]. The calculation results showed that the maximum seismic intensity was VIII, entirely concentrated in Yangbi County. The majority of the intensity VII area was distributed in Yangbi County and a small part extended to sparsely populated areas in Yongping County in the southwest. The densely populated areas of the counties and cities adjacent to Yangbi only suffered a maximum seismic intensity VI, among which the densely populated areas of Eryuan County in the north and Yongping County in the southwest only suffered a seismic intensity V. Damage caused by earthquakes with intensity V–VI are generally considered to be highly limited [42]. Therefore, we estimated that the overall damage due to this earthquake was relatively small. The intensity distribution began from the fault as the center, extended outward in an overall butterfly-like shape, and gradually decayed. The main reasons for this distribution pattern for the seismic intensity are that the fault rupture scale of this earthquake was small, and the fault slip was dominated by strike-slip. Therefore, strong S-wave energy was generated along the fault strike and perpendicular to the fault strike, finally presenting a distribution pattern similar to the double couple point source radiation pattern on the surface. In addition, the fault strike, dip angle and slip angle were about 138°, 73° and –163°, respectively. The fault hanging wall was located in the southwest. Owing to the hanging wall effect of the fault, the energy in the southwest direction was stronger than that in the footwall direction. The asymmetric shape of the specific details for the intensity also reflected the non-uniformity of the surface seismic intensity distribution under the actual complex conditions (i.e., the media and terrain).

### 3.3. Fatality Estimation Results

**Figure 6:**
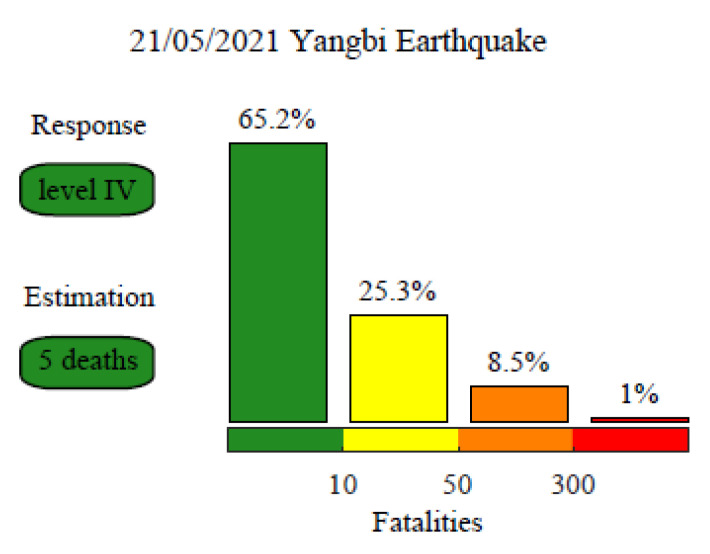
Rapid estimation of the fatalities and earthquake emergency response level for the Yangbi earthquake.

Based on the above seismic intensity distribution data, GIS software (Version 10.9) was used to calculate regional superposition using LandScan population distribution data to obtain population exposure values in different seismic intensity areas. Equations (5) and (7) are then used to estimate the fatalities related to the Yangbi earthquake and the probability of the emergency response levels, as shown in Figure 6. Five fatalities were estimated; the highest probability for the estimated fatality range was 0–10, with a value of 65.2%. According to the post-earthquake emergency response regulations in the NEEP, this event would be defined as a “General Earthquake Disaster Event” with a high probability, corresponding to a Level IV earthquake emergency response. According to the population density distribution, two relatively densely populated cities are located nearby the epicenter: Dali City in the east and Weishan County in the southeast. However, the densely populated areas only suffered from seismic intensity VI. Overall, we considered that the earthquake was relatively minor and did not cause major damage. Finally, we proposed the initiation of a Level IV emergency response plan, using Yangbi County as the main rescue area during earthquake relief. Weishan County, Dali City, Eryuan County, Yunlong County, and Yongping County were considered as secondary concerns. Among them, the northwest area in the two urban areas of Dali and Weishan is the key disaster relief area in the secondary disaster relief.

On 23 May, the Yunnan provincial government reported a final fatality count of three for the Yangbi earthquake (see Data Availability Statement). Even though the estimates deviated from the reported fatalities, the error remained within one order of magnitude, consistent with expectations. In addition, the reported fatalities also occurred within the 0–10 fatality range estimated in this study. In summary, the estimation results for the Yangbi earthquake were ideal, which shows that the fatality estimation method is sufficient to conform to the actual needs of the government for rapidly implementing earthquake emergency response decisions after an earthquake.

## 4. Discussion

**Figure 7:**
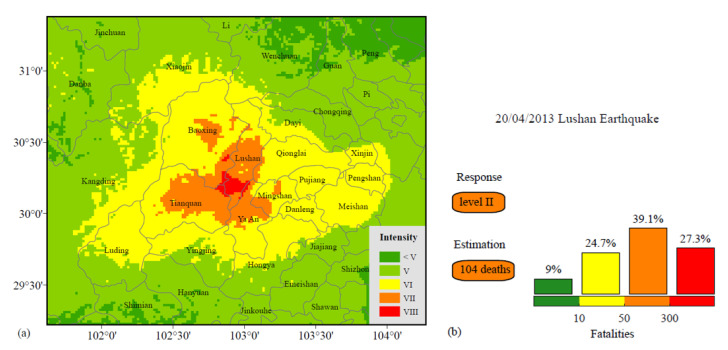
(**a**) The seismic intensity distribution calculated by numerical simulations for the 2013 Ms 7.0 Lushan earthquake. (**b**) The rapid estimation of the fatalities and earthquake emergency response level for the 2013 Ms 7.0 Lushan earthquake.

Even though the Yangbi earthquake is a low-fatality earthquake event, it was selected as a case study here, because it was not among the earthquake events used in the original fatality estimation model. To further verify the effectiveness of this method, we selected the Ms 7.0 Sichuan Lushan earthquake on 20 April 2013 in our estimation model data. This earthquake caused great damage, which resulted in 196 deaths and direct economic losses of more than 10 billion US dollars. The source model used in this case was provided by Wang et al. [43]. The seismic intensity distribution was obtained by strong ground motion simulation, as shown in Figure 7a, where the maximum seismic intensity is VIII in this event. The hardest-hit areas of seismic intensity VII–VIII were concentrated in five cities near the epicenter (Lushan, Baoxing, Tianquan, Mingshan, and Ya An), in which Lushan suffered the most serious damage. The estimated high-intensity areas correspond to the most severely damaged areas reported after the earthquake (see Data Availability Statement). As shown in Figure 7b, the final estimated number of deaths is 104, and the fatality range with the largest probability of this earthquake is 50–300. According to the NEEP, a Level II earthquake emergency response plan should be initiated for this event. In this case, the expected and recorded fatalities have the same magnitude order, and the number of recorded deaths is in the estimated fatality range. If the plan was initiated according to our estimated earthquake emergency response level, it would fully meet the actual needs of this earthquake disaster relief. This reasonable result is significant for the earthquake relief and rescue effort.

The numerical simulation results depended on the source model, velocity medium, terrain, and other data. At present, there are no unified results of other studies for the previously mentioned variables, except terrain, and determining the same is beyond the scope of this paper. Most source models are derived from the inversion of teleseismic waveform data; the maximum frequency of waveforms is usually lower than that of the near-field waveforms. Zhang et al. comprehensively discussed the influence of different frequencies on the numerical simulation results for strong ground motion, based on the Sunway TaihuLight supercomputer (Institute of Computing Technology, Chinese Academy of Sciences, China). The supercomputer is the world’s first supercomputer with a peak performance exceeding 100 PFlops and 1.3 PB memory [44]. They found that the sensitivity of the surface seismic intensity to frequency change is low [45]. Therefore, the accuracy of the simulation results based on teleseismic data in this study was sufficient to meet the requirements of preliminary post-earthquake rapid estimation. In addition, the final fatality estimation results obtained in this study also demonstrated the reliability of the method. High-frequency waveforms generated in the near field could contain more details on sources. However, seismic stations are absent in some areas near epicenters, or the local government does not disclose the data, consequently, making it difficult for researchers to obtain near-field data at the first time. Teleseismic data not only ensure the implementation of this method rapidly after an earthquake but also produce timely estimation results. Further, the numerical intensity and estimated fatalities will be updated with continuous improvements to post-earthquake data collection (e.g., near-source data or more accurate teleseismic data).

The fatality estimation method is an empirical method based on regression analysis. Therefore, the estimated results cannot be used as an accurate prediction for the fatalities of an earthquake. However, the estimated value can be guaranteed to be within one order of magnitude of the actual number of fatalities [12]. In other words, the model can estimate the number of fatalities within a reasonable range. Therefore, the method is appropriate for rapid fatality estimations during a post-earthquake emergency response. Using this method to estimate the probability of each fatality range can provide a scientific quantitative basis to the government and related agencies to activate a specific earthquake emergency response plan based on the NEEP. Regarding the case study of the Yangbi earthquake on 21 May 2021, we determined the number of fatalities in the range of 0–10 (the calculated expected value was 5). Based on the quantitative estimated fatalities and NEEP, we evaluate that this earthquake should be initiated a Level IV earthquake emergency response plan. However, the local government actually initiated a Level II emergency response plan after the earthquake, which overestimated the damage degree of the earthquake and led to unnecessary wastage of resources. The emergency response level estimated by our method was more consistent with the actual mitigation requirements. In addition, the actual number of fatalities was within the estimated range and within an order of magnitude of the expected estimates. Therefore, it can be concluded that our estimation results were consistent with the expectations.

There are some limitations of this study, for example, the uncertainty of each parameter in the fatality estimation model needs to be further determined. The model is currently too simplified. In the future, some parameters that are highly correlated with the fatalities can be considered to further enhance the estimation accuracy. In addition, the far-field data carries fewer high-frequency components than the near-field. In the future, if we can cooperate with earthquake-related institutions in Mainland China to obtain near-field data as soon as possible, the accuracy of the final calculation results will be further increased.

The above result demonstrates the potential of the proposed method to assist the government in a rapid post-earthquake emergency response. In the future, if an earthquake occurs in Mainland China, the application of this method will facilitate rapid estimation of the earthquake fatalities and indicate the appropriate emergency response level. These estimations can provide scientific guidance to the government and relevant institutions for real-time disaster relief applications, such as rapid post-earthquake emergency response decision-making and appropriate relief and resource allocation. Finally, it can reduce additional losses and save more lives.

## 5. Conclusions

Combining a physics-based numerical simulation of earthquake intensity and a statistics-based fatality estimation model, we propose a method for rapidly estimating the number of fatalities and emergency response levels after an earthquake. We successfully applied it to the Ms6.4 Yangbi earthquake in Yunnan on 21 May 2021, and the calculated results are reasonable compared with the actual number of deaths. In the future, if an earthquake occurs in Mainland China, the application of this method will facilitate rapid estimation of the earthquake fatalities and indicate the appropriate emergency response level. These estimations can provide scientific guidance to the government and relevant institutions for real-time disaster relief applications, such as rapid post-earthquake emergency response decision-making and appropriate relief and resource allocation. Finally, it can reduce additional losses and save more lives.

Other similar countries or regions that have experienced many earthquakes can use the method proposed in this study as a reference. Based on the local historical human loss data, the fatality assessment model was constructed using regression analysis, and combined with the open-source GPU numerical simulation program; consequently, a method for timely assessment of fatalities was developed. In areas with few historical earthquakes, information on adjacent areas can be considered to evaluate the fatalities.

## Data Availability

The data consulted for this article can be found from the following websites: (1) Mainland China Composite Damaging Earthquake Catalog available at https://zenodo.org/record/6514359#.Yoh2bhpByUm (accessed on 3 May 2022); (2) National Earthquake Emergency Plan (NEEP) for Mainland China available at http://www.gov.cn/yjgl/2012-09/21/content_2230337.htm (accessed on 21 May 2022); (3) Shuttle Radar Topographic Mission digital elevation dataset provided by the Consultative Group for International Agricultural Research-Consortium for Spatial Information available at https://cgiarcsi.community/data/srtm-90m-digital-elevation-database-v4-1/ (accessed on 21 May 2022); (4) Chinese crust-upper mantle seismic reference model available at http://chinageorefmodel.org (accessed on 13 January 2022); (5) China Seismic Intensity Scale (GB/T 17742-2020) available at http://www.gb688.cn/bzgk/gb/std_list?p.p1=0&p.p90=circulation_date&p.p91=desc&p.p2=GB/T%2017742 (accessed on 21 May 2022); (6) population distribution data provided by LandScan and available at https://landscan.ornl.gov/ (accessed on 13 January 2022); and (7) The Yangbi earthquake reported fatalities from the Yunnan Province government available at http://www.yn.gov.cn/ywdt/zsdt/202105/t20210523_222657.html (accessed on 13 January 2022); (8) The description of the Lushan earthquake disaster available at https://en.wikipedia.org/wiki/2013_Lushan_earthquake (accessed on 21 May 2022). (9) Figure 2 and Figure 3 were created using Generic Mapping Tools [46]. (10) The seismic source data used in this study can be requested from Weimin Wang (personal communication).

## References

[1] Li Y., Zhang Z., Xin D. (2021). A Composite Catalog of Damaging Earthquakes for Mainland China. Seismol. Res. Lett..

[2] Tang B., Chen Q., Liu X., Liu Z., Liu Y., Dong J., Zhang L. (2019). Rapid estimation of earthquake fatalities in China using an empirical regression method. Int. J. Disast. Risk Reduct..

[3] FEMA (2006). HAZUS-MH 2.1 Technical Manual.

[4] Porter K. (2009). Cracking an Open Safe: HAZUS Vulnerability Functions in Terms of Structure-Independent Spectral Acceleration. Earthq. Spectra.

[5] Ceferino L., Kiremidjian A., Deierlein G. (2018). Probabilistic model for regional multiseverity casualty estimation due to building damage following an earthquake. ASCE-ASME J. Risk Uncertain. Eng. Syst..

[6] Kawasumi H. (1954). Intensity and Magnitude of Shallow Earthquakes, Bureau Central Seism. Intern. Ser. A Trav. Sci..

[7] Samardjieva E., Badal J. (2002). Estimation of the expected number of casualties caused by strong earthquakes. Bull. Seismol. Soc. Am..

[8] Jaiswal K., Wald D.J., Hearne M. (2009). Estimating Casualties for Large Earthquakes Worldwide Using an Empirical Approach.

[9] Jaiswal K., Wald D.J. (2010). An empirical model for global earthquake fatality estimation. Earthq. Spectra.

[10] Firuzi E., Hosseini K.A., Ansari A., Izadkhah Y.O., Rashidabadi M., Hosseini M. (2020). An empirical model for fatality estimation of earthquakes in Iran. Nat. Hazards.

[11] Daniell J.E., Khazai B., Wenzel F., Vervaeck A. (2011). The CATDAT damaging earthquakes database. Nat. Hazards Earth Syst. Sci..

[12] Li Y., Xin D., Zhang Z. (2021). A rapid-response earthquake fatality estimation model for mainland China. Int. J. Disast. Risk Re..

[13] (CIESIN) C.f.I.E.S.I.N., (CIAT) C.I.d.A.T. Gridded Population of the World, Version 3 (GPWv3) Data Collection. http://sedac.ciesin.columbia.edu/data/collection/grump-v1.

[14] Tatem A., Linard C. (2011). Population mapping of poor countries. Nature.

[15] Rose A.N., McKee J.J., Urban M.L., Bright E.A., Sims K.M. (2019). LandScan 2018.

[16] Hu Y. (1988). Earthquake Engineering.

[17] Wang D., Wang X., Kou A., Ding X. (2007). Primary study on the quantitative relationship between the typical building structures in western China. Earthquake.

[18] Paolucci R., Gatti F., Infantino M., Smerzini C., Güney Özcebe A., Stupazzini M. (2018). Broadband Ground Motions from 3D Physics-Based Numerical Simulations Using Artificial Neural Networks. Bull. Seismol. Soc. Am..

[19] Infantino M., Mazzieri I., Özcebe A.G., Paolucci R., Stupazzini M. (2020). 3D Physics-Based Numerical Simulations of Ground Motion in Istanbul from Earthquakes along the Marmara Segment of the North Anatolian Fault. Bull. Seismol. Soc. Am..

[20] Stupazzini M., Infantino M., Allmann A., Paolucci R. (2021). Physics-based probabilistic seismic hazard and loss assessment in large urban areas: A simplified application to Istanbul. Earthq. Eng. Struct. Dyn..

[21] Xin D., Zhang Z. (2021). On the Comparison of Seismic Ground Motion Simulated by Physics-Based Dynamic Rupture and Predicted by Empirical Attenuation Equations. Bull. Seismol. Soc. Am..

[22] Tromp J., Komatitsch D., Hjörleifsdóttir V., Liu Q., Zhu H., Peter D., Bozdag E., McRitchie D., Friberg P., Trabant C. (2010). Near real-time simulations of global CMT earthquakes. Geophys. J. Int..

[23] Lee S.-J., Liang W.-T., Cheng H.-W., Tu F.-S., Ma K.-F., Tsuruoka H., Kawakatsu H., Huang B.-S., Liu C.-C. (2014). Towards real-time regional earthquake simulation I: Real-time moment tensor monitoring (RMT) for regional events in Taiwan. Geophys. J. Int..

[24] Wang W., Zhang Z., Zhang W., Yu H., Liu Q., Zhang W., Chen X. (2022). CGFDM3D-EQR: A Platform for Rapid Response to Earthquake Disasters in 3D Complex Media. Seismol. Res. Lett..

[25] Zhang W., Chen X. (2006). Traction image method for irregular free surface boundaries in finite difference seismic wave simulation. Geophys. J. Int..

[26] Zhang W., Zhang Z., Chen X. (2012). Three-dimensional elastic wave numerical modelling in the presence of surface topography by a collocated-grid finite-difference method on curvilinear grids. Geophys. J. Int..

[27] Zhang W., Shen Y., Zhao L. (2012). Three-dimensional anisotropic seismic wave modelling in spherical coordinates by a collocated-grid finite-difference method. Geophys. J. Int..

[28] Sun Y.-C., Ren H., Zheng X.-Z., Li N., Zhang W., Huang Q., Chen X. (2019). 2-D poroelastic wave modelling with a topographic free surface by the curvilinear grid finite-difference method. Geophys. J. Int..

[29] Zhang W., Shen Y., Chen X. (2008). Numerical simulation of strong ground motion for the Ms8.0 Wenchuan earthquake of 12 May 2008. Sci. China Ser. D Earth Sci..

[30] Zhu G., Zhang Z., Wen J., Zhang W., Chen X. (2013). Preliminary results of strong ground motion simulation for the Lushan earthquake of 20 April 2013, China. Earthq. Sci..

[31] Zhang Z., Zhang W., Sun Y., Zhu G., Wen J., Chen X. (2014). Preliminary simulation of strong ground motion for Yutian, Xinjiang earthquake of 12 February 2014, and hazard implication. Chin. J. Geophys..

[32] Zhang Z., Sun Y., Xu J., Zhang W., Chen X. (2014). Preliminary simulation of strong ground motion for Ludian, Yunnan earthquake of 3 August 2014, and hazard implication. Chin. J. Geophys..

[33] Hixon R. (1997). On Increasing the Accuracy of MacCormack Schemes for Aeroacoustic Applications. AIAA Pap..

[34] Cheng J., Grossman M., McKercher T. (2014). Professional CUDA C Programming.

[35] Reuter H., Nelson A., Jarvis A. (2007). An Evaluation of Void-Filling Interpolation Methods for SRTM Data. Int. J. Geogr. Inf. Sci..

[36] Shen W., Ritzwoller M.H., Kang D., Kim Y., Lin F.-C., Ning J., Wang W., Zheng Y., Zhou L. (2016). A seismic reference model for the crust and uppermost mantle beneath China from surface wave dispersion. Geophys. J. Int..

[37] Wang W., He J., Hao J., Yao Z. (2021). Preliminary Result for Rupture Process of May. 21, 2021, M6.4 Earthquake, Dali, China.

[38] Stidham C., Antolik M., Dreger D., Larsen S., Romanowicz B. (1999). Three-dimensional structure influences on the strong-motion wavefield of the 1989 Loma Prieta earthquake. Bull. Seismol. Soc. Am..

[39] Cárdenas-Soto M.n., Chávez-García F.J. (2003). Regional path effects on seismic wave propagation in central Mexico. Bull. Seismol. Soc. Am..

[40] Komatitsch D., Liu Q., Tromp J., Suss P., Stidham C., Shaw J.H. (2004). Simulations of ground motion in the Los Angeles basin based upon the spectral-element method. Bull. Seismol. Soc. Am..

[41] Khan S., Meijde M.v.d., Werff H.v.d., Shafique M. (2020). The impact of topography on seismic amplification during the 2005 Kashmir earthquake. Nat. Hazards Earth Syst. Sci..

[42] (2020). The Chinese Seismic Intensity Scale.

[43] Wang W., Hao J., Yao Z. (2013). Preliminary result for rupture process of Apr. 20, 2013, Lushan Earthquake, Sichuan, China. Chin. J. Geophys..

[44] Fu H., Liao J., Yang J., Wang L., Song Z., Huang X., Yang C., Xue W., Liu F., Qiao F. (2016). The Sunway TaihuLight supercomputer: System and applications. Sci. China Inf. Sci..

[45] Zhang W., Zhang Z., Fu H., Li Z., Chen X. (2019). Importance of Spatial Resolution in Ground Motion Simulations With 3-D Basins: An Example Using the Tangshan Earthquake. Geophys. Res. Lett..

[46] Wessel P., Smith W.H.F. (1998). New improved version of Generic Mapping Tools released. Eos Trans. Am. Geophys. Union.

